# Influence of long-term supplementation of a formulated botanical blend on growth performance and carcass traits in feedlot steers

**DOI:** 10.5713/ab.24.0125

**Published:** 2024-06-25

**Authors:** Thiago Lauro Maia Ribeiro, Becca B. Grimes Francis, Erin R. DeHaan, Cassidy R. Ross, Justin J. Delver, Forest L. Francis, Jeff S. Heldt, Emma H. Wall, Warren C. Rusche, Zachary K. Smith

**Affiliations:** 1 Department of Animal Science, South Dakota State University, Brookings, SD 57007, USA; 2 Selko® USA, Indianapolis IN 46241, USA; 3 Nutreco Exploration, Nutreco, P.O. Box 299, 3800 AG Amersfoort, The Netherlands

**Keywords:** Beef, Feedlot, Finishing Phase, Phytogenic

## Abstract

**Objective:**

The objective of this study was to determine if a formulated blend of capsicum oleoresin, clove essential oil, and garlic essential oil (Fytera Advance - Selko USA, Indianapolis IN, USA; CCG) influences measures of cattle growth, efficiency, or carcass traits, during the finishing phase in steers fed a concentrate-based diet.

**Methods:**

Charolais×Angus steers (n = 96; initial shrunk body weight [BW] = 391±34.0 kg) were used in a 144-d (16 February 2023 to 9 July 2023) finishing feedlot experiment in Brookings, SD, USA. Steers were individually weighed and allotted to one of 14 pens (6 to 7 steers; 7 pens/treatment) in a randomized complete block design and randomly assigned to 1 of 2 treatments: control diet without the test product (CON) or a diet including CCG at 500 mg/steer daily (CCG). Steers were fed twice daily, and bunks were managed according to a slick bunk system.

**Results:**

There were no differences (p≥0.10) in any growth performance outcomes from d 1 to 35, 36 to 70, or 71 to 98. From d 99 to 144 steers from CCG tended to have 5% greater average daily gain (p = 0.09) and 8% improved feed conversion (G:F) (p = 0.01). No differences (p≥0.15) were noted for cumulative growth performance measures. No differences were noted for any carcass measurements or categorical carcass outcomes, nor lung or liver health outcomes (p≥0.15).

**Conclusion:**

The use of CCG had no influence on cumulative growth performance responses. However, the use of CCG improved G:F during the late feeding period.

## INTRODUCTION

Botanicals (essential oils [EO], plant extracts) contain secondary metabolites produced by medicinal plants to elicit protective effects and defense mechanisms for the host plant [1]. Because of their anti-microbial activity at high doses, various botanicals have been explored for their potential to modulate the rumen environment, by either promoting or inhibiting specific microbial populations [1]. Subsequent studies have revealed the antimicrobial and anti-inflammatory properties, in which, can improve feed efficiency by modulating ruminal and host metabolism [2]. However, most research investigating the effect of EO on rumen fermentation has been conducted *in vitro*, and *in vivo* studies demonstrating this effect on beef cattle are limited [3].

Host-mediated effects of botanicals in ruminants have been established. For example, capsaicinoids from chili peppers—when fed at low doses—can decrease inflammation in dairy cows challenged with lipopolysaccharide (LPS), and can influence glucose metabolism and nutrient partitioning [4]. Moreover, these responses carry implications for beef cattle, as finishing diets trigger various physiological changes. The inclusion of highly fermentable carbohydrates leads to a decline in ruminal pH, resulting in the accumulation of short-chain fatty acids and lactate in the rumen. This, in turn, may cause damage to the ruminal epithelium and impair tissue function, potentially allowing the translocation of pathogenic substances from the rumen into the bloodstream [5]. The lysis of gram-negative bacteria during ruminal acidosis elevates LPS concentration in the ruminal fluid. Given that LPS is a potent proinflammatory endotoxin in the circulatory system, it has the potential to induce leaky gut and cause liver abscessation, thereby diminishing overall performance [5]. However, available data indicate that the supplementation of EOs in the diet increases oxidative stress in plasma while mitigating acute phase responses induced by LPS in cattle [6].

Phenylpropene eugenol, a predominant compound found in clove EO, exerts its influence on rumen microbiota by inhibiting glycolytic enzyme activity, thereby impeding the microbe’s ability to utilize intracellular glucose [7]. This disruption in microbial metabolism can lead to significant alterations in the rumen ecosystem. Moreover, clove EO demonstrates notable anti-inflammatory properties within the gastrointestinal tract and other tissues, potentially improving overall health [7]. Furthermore, *in vitro* studies have highlighted its efficacy as an anthelmintic agent against *Haemonchus contortus* in sheep [8], underscoring its multifaceted potential as a natural remedy for gastrointestinal health and parasite control in livestock.

Garlic essential oil (GEO) has several sulfur compounds which have activities against a wide range of Gram positive and Gram negative bacteria, including pathogenic bacteria [9]. Additionally, when GEO was added to an *in vitro* culture fermentation, methane production decreased by 19.5% without altering digestibility, where the rumen methanogenesis was accompanied by a decrease in the acetate: propionate ratio [9]. Thus, GEO can improve immune function; parasite resistance and growth performance [10].

Monensin is an antibiotic ionophore approved as a feed additive by the FDA in 1975. The mood of action of monensin consists in selectively target the gram-positive microorganisms, disrupting the ion concentration gradient across specific microbial membranes [11]. The current prohibition on ionophore supplementation within the European Union, coupled with a potential growing trend in other regions. Therefore, increasing efforts to explore ‘organic’ alternatives to conventional antibiotic supplements [11]. Essential oils represent a possible alternative for decrease the use of antibiotics. However, monensin is still fed to a large proportion of finishing cattle fed in both North and South America, and Australia. Yet, studies testing the use of monensin and EOs combined are limited. Despite extensive work in various livestock species, studies on host-mediated responses of beef cattle to botanicals—and the associated effects on performance—are limited. Therefore, the objective of this study was to determine if a blend of capsicum oleoresin, clove, and GEOs influences measures of cattle growth performance and efficiency, and carcass traits, during the finishing phase in feedlot steers fed a concentrate-based diet.

## MATERIALS AND METHODS

### Use of animal subjects

The animal care and handling protocols used in this study were approved by the South Dakota State University Institutional Animal Care and Use Committee #2312-011E. This study was conducted at the Ruminant Nutrition Center (RNC) located in Brookings, South Dakota, between 16 February 2023 and 9 July 2023.

### Animal description and processing

Charolais×Angus steers (n = 96; initial shrunk body weight [BW] = 391±34.0 kg) were used in a 144-d (16 February 2023 to 9 July 2023) finishing period experiment. Steers were tagged, vaccinated against viral respiratory (Bovi-Shield Gold 5; Zoetis, Parsippany NJ, USA) and clostridia pathogens (Ultrabac 7/Somubac; Zoetis, USA) according to label instructions, and poured with moxidectin (Cydectin; Elanco, Indianapolis, IN, USA) the previous November. All steers were implanted on d 35 (110 d before harvest) with a 200 mg trenbolone acetate and 28 mg estradiol benzoate implant (Synovex Plus; Zoetis, USA). All implants were checked on d 70 and no implant anomalies were noted.

### Experimental design and treatments

This study used 7 replicate pens per treatment and each pen contained 6 to 7 steers (48 steers/treatment). Each pen was assigned to one of two dietary treatments in a randomized complete block design (RCBD), where location within the feedlot was considered the blocking factor. Dietary treatments included: i) non-supplemented control (CON); ii) supplemented with 500 mg/steer daily of a formulated blend of capsicum oleoresin, and natural, full-spectrum clove and GEOs (Fytera Advance - Selko USA, Indianapolis IN, USA; CCG). According to non-published data, steers that received 500 mg/d had improved serum immunological and growth related metabolite levels when compared to steers that received 125 and 250 mg/d. The dietary treatment was included in a soybean hulls carrier and offered in replacement of dry-rolled corn at a rate of 0.454 kg/steer daily (as-fed basis).

### Dietary management

All steers were fed twice daily (0800 h and 1400 h) in equal proportions, and bunks were managed for *ad libitum* access (slick bunk management approach) to feed with minimal day to day variation in the amount of feed not consumed being the primary target for feed calls (Table 1). The slick bunk management is the most common practice in the feedlot sector related to bunk management, it promotes enhancements in dry matter intake (DMI) over the feeding period. Additionally, it is thought to mitigate overconsumption tendencies, thereby lowering the risk of metabolic disorders [12]. Pens were 7.6×7.6 m concrete surface with 7.6 m of linear bunk space and equipped with a heated, continuous flow concrete waterer.

The liquid supplement provided to the diet (dry matter [DM] basis) 30 g of Monensin/907 kg (Elanco Animal Health, Greenfield, IN, USA) and hydroxy-trace minerals content were added to the supplement to provide the diet Co at 0.2 mg/kg, Cu at 10 mg/kg, Mn at 20 mg/kg, and Zn at 90 mg/kg, all other nutrients were formulated to meet the nutrient requirements for growing and finishing beef steers [13].

Feed was manufactured in a stationary mixer (2.35 m^3^; 4 or 3 pens fed per batch), all ingredients were added into the mixer to the nearest pound, and feed was delivered to each pen separately (weighed out of the stationary mixer to the nearest pound) into a feed delivery wagon that was not mounted on load cells. Batching sequence was: CON (4 pens), CCG (4 pens), CON (3 pens), and finally CCG (3 pens). Following each batch of feed, long stem grass hay (~2 kg) was added to the mixer and used to flush out all residual feed remaining in the mixer. Mixing of the following batch did not occur until the scale head indicated 0 to 0.45 kg.

Feedstuff samples were taken weekly and analyzed for DM content. Samples were composited monthly for proximate analysis using AOAC procedures (crude protein [CP], neutral detergent fiber [NDF], acid detergent fiber [ADF], and ash). Weekly ingredient samples were stored in a freezer at −20°C until nutrient analyses were completed. After weekly DM determination (method no. 935.29), weekly samples from each ingredient were composited by month and analyzed for N (method no. 968.06; Rapid Max N Exceed; Elementar, Mt. Laurel, NJ, USA), and ash (method no. 942.05) content [14,15]. Distillers grains plus solubles samples were analyzed for ether extract content using an Ankom Fat Extractor (XT10; Ankom Technology, Macedon, NY, USA). Percentages of ADF and NDF were assumed to be 3 and 9 percent for dry-rolled corn [16]. Analysis of ADF and NDF composition for all other feeds was conducted as described by Goering and Van Soest [17]. Diets presented in Table 1 are actual DM diet composition, actual nutrient concentrations, and tabular energy values [16]. All diet changes were made based upon availability of the feed ingredients at each period.

### Growth performance calculations

Steers were individually weighed on d −7, 1, 35, 70, 98, and 144. Cumulative growth performance was calculated on live and carcass-adjusted basis. All live BW measures used were pencil shrunk 4% to account for gastrointestinal tract fill and carcass-adjusted final BW was calculated from hot carcass weight (HCW) divided by 0.625. Average daily gain (ADG) was determined as the difference between final and initial BW divided by days on feed (144 d). Dry matter intake was tabulated at weekly intervals and summarized by interim period. Feed conversion ratio was calculated using ADG divided by DMI.

### Carcass, liver and lung evaluation

Steers were marketed and harvested at a commercial abattoir with treatment blinded personnel determined that 60% of the population has sufficient fat cover to grade USDA Choice. Steers were loaded onto trucks, shipped 230 km, and harvested the following. Liver abscess prevalence and severity was determined by a trained technician using the Elanco system as: normal (no abscesses), A– (1 or 2 small abscesses or abscess scars), A (2 to 4 well organized abscesses less than 2.54 cm, diameter), or A+ (1 or more large active abscesses greater than 2.54 cm, diameter with inflammation of surrounding tissue). Individual lungs were evaluated according to the scale described by Mayer et al [18], 0 = normal; 1 = presence of mycoplasma-like lesion >15%; 2 = plural adhesions, a portion of the lung missing, or a combination of these affecting <25% of lung tissue; 3 = plural adhesions, a portion of the lung missing, or a combination of these affecting >25% to <50% of lung tissue; 4 = plural adhesions, a portion of the lung missing, or a combination of these affecting >50% to <75% of lung tissue; 5 = plural adhesions, a portion of the lung missing, or a combination of these affecting >75% of lung tissue. Video image data was obtained from the plant for rib eye area, rib fat, kidney-pelvic-heart fat, calculated USDA Yield Grade and USDA marbling scores. Dressing percentage was calculated as HCW/(final BW×0.96). Estimated empty body fat (EBF) percentage and AFBW were calculated from observed carcass traits [19], and proportion of closely trimmed boneless retail cuts from carcass round, loin, rib, and chuck was determined according to the equation described by Murphey et al [20].

### Management of pulls and removals

All steers pulled from their home pen for health evaluation were monitored in individual hospital pens prior to being returned to their home pens. When a steer was moved to a hospital pen the appropriate amount of feed from the home pen was removed and transferred to the hospital pen. If a steer in the hospital returned to their home pen, the feed was credited to the home pen. If a steer did not return to their home pen, all feed that is delivered to the hospital pen was deducted from the feed intake record for that particular pen back to the date the steer was hospitalized. One steer was removed from the experiment for reasons not related to dietary treatment and was excluded from growth performance calculations.

### Estimation of dietary net energy from growth performance

Growth performance (live-basis) was used to calculate performance-based dietary net energy (NE) in order to determine efficiency of dietary NE utilization. The performance-based dietary NE was calculated from daily energy gain (EG; Mcal/d): EG = ADG^1.097^×0.0557W^0.75^, where W is the mean equivalent shrunk BW (kg; NRC [13]) from median feeding shrunk BW and final BW at 28% estimated empty body fatness (AFBW) calculated as: (median feeding shrunk BW×(478/AFBW), kg; NRC [13]). Maintenance energy (EM) was calculated by the equation: EM = 0.077×median feeding shrunk BW^0.75^. Dry matter intake is related to energy requirements and dietary NEm (Mcal/kg) according to the following equation: DMI = EG/(0.877NEm–0.41), and can be resolved for estimation of dietary NEm by means of the quadratic formula $\text{x}=\left(-\text{b}\pm \sqrt{\left({\text{b}}^{2}-4\text{ac}\right)}\right)/2\text{c}$, where a = −0.41EM, b = 0.877EM+0.41DMI+EG, and c = −0.877DMI [21]. Dietary NEg was derived from NEm using the following equation: NEg = 0.877NEm–0.41 [22].

### Climatic variables and temperature humidity index calculation

Ambient temperature and relative humidity information were obtained from an on-site weather station (Vantage Pro2; Davis Instruments Corporation, Hayward, CA, USA). Temperature humidity index (THI) was calculated based on Hahn et al [23] using the formula, THI = 0.8×ambient temperature+([% relative humidity/100]×[ambient temperature–14.4])+46.4. Where a THI greater than 72 indicates mild stress, while a THI above 89 indicates severe stress [24].

### Statistical analysis

Growth performance, carcass traits, and efficiency of dietary energy utilization were analyzed as a RCBD using the GLIMIX procedure of SAS 9.4 (SAS Inst. Inc., Cary, NC, USA) with pen as the experimental unit. The model included the fixed effect of dietary treatment and block was considered a random variable. Distribution of USDA Yield and Quality grade, as well as liver abscess and lung lesion severity and prevalence data were analyzed as multinomial distributions using the GLIMMIX procedure of SAS 9.4 to identify differences in the distributions among treatments. Individual steer served as the experimental unit for categorical outcome data and the same fixed and random effects in the model as described previously were used. The model specified a solution function for the multinomial response, with the number of animals slaughtered identified in the denominator. Least squares means were generated using the LSMEANS statement of SAS 9.4. Treatment effects were evaluated by the use pairwise comparisons PDIFF option in SAS 9.4. An α of 0.05 was used to determine significance and an a of 0.06 to 0.10 was considered a tendency.

## RESULTS

Growth performance outcomes are located in Table 2 and carcass outcomes are located in Table 3. Climatic variables for each period are listed in Table 4. There were no differences noted for any growth performance measures from d 1 to 35, 36 to 70, or 71 to 98 (p≥0.25). From d 99 to 144 steers from CCG tended to have 5% greater ADG (p = 0.09) and had improved feed efficiency by 8% (p = 0.01). However, there were no statistical differences on applied energetics (p>0.68), meaning that the steers performed as expected. No differences (p≥0.15) were noted for cumulative growth (live or from HCW/0.625). Finally, no differences were noted for any carcass traits or categorical carcass outcomes, lung nor liver health outcomes (p≥0.15).

## DISCUSSION

Commonly, multiple EO are used as a combination to obtain maximum improvements in growth performance and health benefits. Additionally, very limited research is available on the potential synergies among them [25]. Furthermore, monensin sodium is an ionophore that might influence responses associated with the use of various botanical compounds of blends of botanical compounds.

In the present study, the inclusion of CCG in the diet did not improve growth performance from d 1 to d 98, in agreement with Gouvêa et al [26], which fed a blend of EO composed of cinnamaldehyde, clove EO, and capsicum oleoresin for steers confined in a feedlot system. Additionally, Eidsvik et al [27] observed no difference in final body weight, intake, gain, nor efficiency when feedlot steers were supplemented with rumen protected capsicum oleoresin at 77, 100, 250, or 322 mg/steer daily compared to control steers. However, from d 99 and d 144 of the present trial, during the late feeding period steers were subjected to high environmental heat load, presented a tendency for a greater ADG and improved feed conversion was observed.

Greater feed efficiency from d 99 to 144, coincided with increased summertime temperatures (Table 4); notably, the daily maximal THI surpassed 72 for 13 consecutive days between d 99 and d 144. Under such ambient conditions, there’s a significant likelihood of compromised energy intake, consequently impacting growth performance adversely [28].

Since daily weight gain is strongly associated with energy intake, variation in ADG can be interpreted as a reflection of variation in DMI [29]. These results are in line with the effect of capsicum oleoresin on the fractionation of feed intake pattern in feedlot cattle fed high concentrate diets [29]. Moreover, Oguey et al [30] supplemented capsicum oleoresin to feedlot cattle in a commercial system during summer, resulted in a tendency for lower daily variation of DMI for supplemented group compared to control (p<0.06), suggesting that capsicum oleoresin induced a more homogeneous DMI. Additionally, EO can change the volatile fatty acids profile in the rumen resulting in a lower molar proportion of acetate and a higher proportion of propionate and butyrate [31]. Since propionate is a precursor in gluconeogenesis and provides more energy to the animal, even when dry matter intake is reduced, the ruminant can be more efficient [31]. These findings are comparable to studies showing that EO can increase feed efficiency.

Potentially, capsaicin can be used during heat stress events due to the activation of chemo- and heat-sensitive receptors, which in mammals induces heat-loss response [32]. Additionally, heat stress in beef cattle is known to induce oxidative stress and inflammation, leading to the impairment of the rumen epithelial barrier and an increased risk of rumen acidosis [33]. Damage to the ruminal epithelium during episodes of rumen acidosis impairs tissue function, thereby enhancing the risk of pathogenic substances migrating into the bloodstream [33]. Bacteria and bacterial endotoxins (LPS) are able to translocate into the bloodstream in these situations, potentially affecting animal health and production due to the inflammation [33]. The EO contained in the botanical blend fed in the present study have the potential to mitigate the inflammatory response, thereby enhancing the likelihood of improved performance compared to the CON. This improvement could be attributed to the health benefits of EO, which save energy that would otherwise be expended on inflammation, allowing it to be redirected towards growth.

In preceding studies, it has been evidenced that *Escherichia coli* (*E. coli*) emerges as a prominent livestock pathogen, implicated in a spectrum of livestock pathologies, including mastitis, ruminal acidosis, and leaky gut syndrome [5,34]. Respiratory and digestive diseases are prevalent and can lead to marked decreases in performance throughout the growth and finishing period. In this regard, Liu et al [35] reported that some botanicals were protective against both Porcine reproductive and respiratory syndrome virus and infection with pathogenic *E. coli*. In theory, the EOs provided during this trial may have contributed to the improvement in late feeding period performance, not only by mitigating heat stress but also by potentially reducing inflammation. However, the duration of the experimental period, the level of concentrate in the diet, and the dose of EOs appear to be factors contributing to the variability of the effects observed in beef cattle supplemented with EOs [36].

Differing from the present trial, when clove EO was fed at 3.5 or 7.0 g/daily to young crossbred bulls, a linear response was observed with increase dosage of clove EO for ADG, DMI, and final BW [37]. Additionally, when a rumen protected blend composed of clove EO and rosemary EO was fed at 4.0 g/heifers daily, there were improvements in ADG, DMI, and feed conversion (G:F) compared to control (p< 0.05); However, no difference was observed for final BW or carcass traits [38], in which is similar to the present trial. Additionally, feeding increasing doses of rumen protected capsicum oleoresin [27] or a blend of cinnamaldehyde, clove EO, and capsicum oleoresin Gouvêa et al [26] to feedlot steers did not affect any carcass characteristics nor liver abscess outcome in feedlot steers compared to the non-supplemented group [26,27]. Canbolat et al [39] fed Kivircik lambs and noted detrimental impacts associated with escalating (0 to 1.2 g/kg of DM) doses of GEO on total weight gain and ADG, however, there were no statistical significance on the final BW, DMI, or G:F. Feeding a GEO to lambs, Chaves et al [40] did not observe any difference on any growth performance outcome or carcass characteristics compared to control group.

## CONCLUSION

The use of CCG showed no significant impact on cumulative growth performance responses, carcass traits, or outcomes related to the liver and lungs, except during the period between day 99 to 144, where a tendency for greater ADG and improved G:F was observed. However, the length of feeding period, length and concentration of EO supplemented, and environmental conditions can be factors that might affect the growth performance responses in ruminants. Additional experiments are necessary to determine the optimal dosage of CCG and the most effective timing and/or duration for its administration to provide optimized efficacy to improve performance of beef cattle.

## Figures and Tables

**Table 1:** Actual diet formulation and nutrient composition fed to steers during the course of the 144 d experiment^1),2)^ (all values on a dry matter basis)

Items	d 1 to 28	d 29 to 42	d 43 to 122	d 123 to 144
High-moisture ear corn (%)	77.9	48.5	14.5	13.9
Dried distillers grains plus solubles (%)	17.7	18.1	19.2	19.3
Dry rolled corn (%)	0.0	28.8	16.4	56.2
High moisture corn (%)	0.0	0.0	39.3	0.0
Grass hay (%)	0.0	0.0	5.8	5.8
Liquid supplement^3)^ (%)	4.4	4.6	4.8	4.8
Nutrient composition				
Crude protein (%)	14.55	14.95	15.63	15.41
Neutral detergent fiber (%)	20.58	17.62	18.12	18.02
Acid detergent fiber (%)	9.50	8.16	7.75	7.45
Ash (%)	4.98	4.90	5.55	5.48
Ether extract (%)	4.21	4.00	3.77	3.79
Net energy for maintenance (Mcal/kg)	1.98	2.05	2.08	2.07
Net energy for gain (Mcal/kg)	1.34	1.39	1.40	1.39

1) All diet changes were made based upon ingredient availability.

2) The dietary treatment was included in a soybean hulls carrier and offered in replacement of high moisture corn during the first 28 days, and for the reaming periods of the study soybean hulls replaced dry-rolled corn at a rate of 0.454 kg/steer daily (as-fed basis).

3) Liquid supplement contained (DM basis): 44.46% CP, 38.78% non-protein nitrogen, 0.90 Mcal/kg of NEm, 0.57 Mcal/kg of NEg, 0.90% ether extract, 16.52% total sugars, 50.77% ash, 11.00% calcium, 0.38% P, 7.07% K, 0.13% Mg, 6.00% NaCl, 3.54% Na, 0.41% S, 4.30 ppm Co, 200.00 ppm Cu, 12.11 ppm I, 615.2 mg/kg EDDI, 525.35 ppm Fe, 404.93 ppm Mn, 2.93 ppm Se, 1,800 ppm Zn, 44,429 IU/kg vitamin A, 444 IU/kg vitamin E, and 585.37 g/907 kg monensin sodium.

**Table 2:** Growth Performance of finishing beef steers

Items	Treatments^1),2)^		SEM	p-value

	CON	CCG		
Steers (n)	48	48	-	-
Pens (n)	7	7	-	-
Initial BW (kg)	391	391	1.4	0.74
Initial to d 35				
d 35 BW (kg)	450	449	5.8	0.61
ADG (kg/d)	1.70	1.66	0.131	0.58
DMI (kg/d)	9.45	9.51	0.135	0.30
G:F	0.179	0.174	0.0060	0.44
d 36 to d 70				
d 70 BW (kg)	515	513	6.0	0.42
ADG (kg/d)	1.84	1.81	0.132	0.66
DMI (kg/d)	10.66	10.55	0.322	0.48
G:F	0.173	0.172	0.0067	0.82
d 71 to d 98				
d 98 BW (kg)	561	564	8.4	0.46
ADG (kg/d)	1.75	1.73	0.168	0.78
DMI (kg/d)	11.12	10.99	0.395	0.49
G:F	0.158	0.157	0.0063	0.90
d 99 to d 144				
d 144 BW (kg)	640	641	10.4	0.78
ADG (kg/d)	1.64	1.74	0.101	0.09
DMI (kg/d)	10.95	10.72	0.241	0.81
G:F	0.150	0.162	0.0029	0.03
Initial to d 144				
ADG (kg/d)	1.72	1.73	0.064	0.72
DMI (kg/d)	10.55	10.45	0.174	0.74
G:F	0.164	0.165	0.0030	0.71
Initial to d 144				
HCW/0.625 (kg)	663	668	8.9	0.28
ADG (kg)	1.89	1.92	0.056	0.23
DMI (kg)	10.55	10.45	0.174	0.74
G:F	0.179	0.183	0.0029	0.29
Applied energetics^3)^				
NEm (Mcal/kg)	2.07	2.08	0.941	0.73
NEg (Mcal/kg)	1.40	1.40	0.824	0.73
O/E NEm	1.00	1.01	0.010	0.68
O/E NEg	1.01	1.01	0.014	0.77

SEM, standard error of the mean; BW, body weight; ADG, average daily gain; DMI, dry matter intake; G:F, feed conversion; HCW, hot carcass weight; NEm, net energy for maintenance; NEg, net energy for gain; O/E NEm, observed/expected net energy for maintenance; O/E NEg, observed/expected net energy for gain.

1) Dietary treatments included: i) fed no capsicum oleoresin, plus clove and garlic essential oil blend (CON); ii) fed capsicum oleoresin, plus clove and garlic essential oil blend at 500 mg/steer daily (Fytera Advance - Selko USA, Indianapolis IN, USA; CCG).

2) A 4% pencil shrink was applied to all BW measures to account for digestive tract fill.

3) Calculated from cumulative live growth (shrunk 4%).

**Table 3:** Carcass trait responses in finishing beef steers

Items	Treatments^1)^		SEM	p-value

	CON	CCG		
Steers, n	48	48	-	-
Pens, n	7	7	-	-
Carcass traits				
Hot carcass weight (kg)	415	418	2.5	0.28
Dressing percentage (%)^2)^	64.83	65.17	0.279	0.27
Ribeye area (cm sq.)	91.94	93.81	0.156	0.12
Rib fat (cm)	1.35	1.37	0.020	0.79
Marbling^3)^	482	487	15.3	0.73
Yield grade	3.24	3.19	0.092	0.60
Retail yield (%)	49.56	49.67	0.188	0.57
Estimated empty body fat (EBF, %)^4)^	30.72	30.77	0.305	0.88
Final body weight adjusted to 28% EBF (kg)^4)^	605	609	16.1	0.64
Yield grade (%)				
1	2.1	6.3	-	0.13
2	38.3	45.8		
3	53.2	45.8		
4	6.4	2.1		
Quality trade (%)				
Select	15.2	10.4	-	0.90
Low choice	47.8	50.0		
Upper choice	37.0	37.5		
Prime	0.0	2.1		
Lung score (%)				
0	83.0	91.6	-	0.27
1	17.0	4.2		
2	0.0	2.1		
3	0.0	2.1		
Liver outcomes (%)				
Normal	100.0	97.9	-	0.99
A−	0.0	0.0		
A	0.0	0.0		
A+ or Greater	0.0	2.1		

SEM, standard error of the mean; HCW, hot carcass weight.

1) Dietary treatments included: i) fed no capsicum oleoresin, plus clove and garlic essential oil blend (CON); ii) fed capsicum oleoresin, plus clove and garlic essential oil blend at 500 mg/steer daily (Fytera Advance - Selko USA, Indianapolis IN, USA; CCG).

2) Calculated as: (HCW/final BW shrunk 4%)×100.

3) Small00 = 400.

4) Calculated from Guiroy et al [19].

**Table 4:** Ambient temperature, mean relative humidity, and temperature-humidity index for each period of the experiment^1)^

Period	DOF	Mean *T_a_* (°C)	Mean RH (%)	Mean THI	Days THI >72	Wind speed, KPH	Total precipitation (cm)
2/16/2023 to 3/22/2023	1 to 35	−6.7	90.8	21.9	0	6.7	1.35
3/23/2023 to 4/26/2023	36 to 70	−3.0	87.6	28.6	0	6.7	0.39
4/27/2023 to 5/17/2023	71 to 98	-	-	-	-	-	-
5/18/2023 to 7/9/2023	99 to 144	21.6	70.9	68.4	13	5.8	8.2
Average	-	8.6	80.0	47.2	-	6.3	9.94 (total)

DOF, days on feed; *T_a_*, ambient temperature; RH, mean relative humidity; THI, temperature-humidity index; KPH, kilometers per hour.

1) A weather station failure occurred for the period from 4/27 to 5/17/2023.

## Data Availability

Data can be made available with reasonable request to Z.K.S.
